# *Adiantum shastense*, a new species of maidenhair fern from California

**DOI:** 10.3897/phytokeys.53.5151

**Published:** 2015-07-21

**Authors:** Layne Huiet, Martin Lenz, Julie K. Nelson, Kathleen M. Pryer, Alan R. Smith

**Affiliations:** 1 Department of Biology, Duke University, Durham, NC 27707; 2 USDA Forest Service, Shasta-Trinity National Forest, 3644 Avtech Parkway, Redding, CA 96002; 3 1001 Valley Life Sciences Building, # 2465, University Herbarium, University of California, Berkeley, CA 94720-2465

**Keywords:** Shasta Lake, maidenhair, Shasta snow wreath, new species

## Abstract

A new species of *Adiantum* is described from California. This species is endemic to northern California and is currently known only from Shasta County. We describe its discovery after first being collected over a century ago and distinguish it from *Adiantum jordanii* and *Adiantum capillus-veneris*. It is evergreen and is sometimes, but not always, associated with limestone. The range of *Adiantum shastense* Huiet & A.R.Sm., **sp. nov.**, is similar to several other Shasta County endemics that occur in the mesic forests of the Eastern Klamath Range, close to Shasta Lake, on limestone and metasedimentary substrates.

## Introduction

The genus *Adiantum* L. (Pteridaceae) is found worldwide mostly in the tropics and subtropics, but about ten percent of species (of a total of ca. 225 spp.) are found in temperate regions. The majority of these occur in Asia but several are found in North America. There are nine species of *Adiantum* in the continental United States and Canada and eight of the nine are native (Paris 1993). Three are of tropical origin, occurring in restricted ranges that are at their northern most limits. The remaining five species occur solely in temperate regions, with four having a broad geographic range: *Adiantum pedatum* L., *Adiantum capillus-veneris* L., *Adiantum aleuticum* (Rupr.) C.A.Paris and *Adiantum jordanii* Müll.Hal. *Adiantum pedatum* and *Adiantum capillus-veneris* are distributed beyond North America (Paris 1993); *Adiantum capillus-veneris* is the most wide-ranging and occurs on six continents, whereas *Adiantum pedatum* occurs widely in both North America and Asia. Cytological data for different geographic localities of these two species reveal differing chromosome numbers (diploids, tetraploids, dysploids), suggesting that they may both be species complexes (Löve and Löve 1997, Nakato and Kato 2005, Wagner 1963).

Of the four wide-ranging species, three *Adiantum capillus-veneris*, *Adiantum aleuticum* and *Adiantum jordanii* occur in California and none of these is endemic. They all are found in at least 30% of the counties, and their distributions span the entire state. *Adiantum aleuticum* is easily recognized by its distinct pseudopedate laminar morphology, while *Adiantum jordanii* has a laminar architecture that is more similar to *Adiantum capillus-veneris*; however, the two are not closely related (Huiet et al. unpublished). Juvenile and sterile forms of these taxa can sometimes be difficult to distinguish.

While investigating *Adiantum capillus-veneris* populations in California as part of a worldwide molecular phylogenetic study of the genus, a new endemic species was discovered. Here we describe this new taxon and discuss its remarkable discovery after it was first collected over 100 years ago.

## Methods

Chromosome material of young sporangia was field-fixed in ethyl alcohol:acetic acid (3:1). Spore mother cells were stained with acetocarmine, and, using standard squash techniques, examined under a compound microscope. Meiotic cells were examined at diakinesis, metaphase I, and normal pairing of homologous chromatids was seen. The voucher is listed under paratypes.

## Taxonomy

### Adiantum shastense

Taxon classificationPlantaePolypodialesPteridaceae

Huiet & A.R.Sm. sp. nov.

urn:lsid:ipni.org:names:77148382-1

Figs 1 , 2

#### Diagnosis.

*Adiantum shastense* is similar to *Adiantum jordanii* in having dark brown to purplish brown rhizome scales and 2–3-pinnate laminae. It differs by being persistent and green throughout the summer, and does not die back as does *Adiantum jordanii*. *Adiantum shastense* can be distinguished from *Adiantum capillus-veneris* by the darker rhizome scales, the rhomboid shape of the pinnulets, and the often, glaucous bluish green color of the laminae.

#### Type.

**UNITED STATES, California**: Shasta County, north side of Lake Shasta. McCloud River arm, along Gilman Road, just W of intersection with Old Mill Road where Fall Creek intersects Gilman Road. 40°51.517200'N, 122°18.835800'W, 1222 ft, 14 May 2014, *Layne Huiet, Alan Smith, Joan Smith, Ellen Dean & Martin Lenz 162* (holotype: UC2030515!; isotypes: CAS!, DAV!, DUKE!, MO!, NY!, US!)

#### Description.

Rhizomes short-creeping or ascending, usually buried in loose soil, 2–4 mm in diameter, sometime branching; stipes clustered, up to 10 fronds per 1 cm of rhizome length; rhizome scales (and those at stipe base) castaneous to dark brown, lustrous, concolorous, ovate to lanceolate, attenuate at tips, 1.5–5 × 0.3–0.6 mm, margins entire; fronds clustered, mostly (18–)30–60 cm long, arching, persisting (remaining green) through summer, fall, and into winter; older dead fronds remaining attached to rhizome behind new growth; stipes castaneous to atropurpureous, becoming blackish with age, sublustrous, sometimes slightly glaucous (especially proximally), terete, each with a single vascular bundle at bases, (10–)20–30 cm long, (0.6–)0.8–2.0 mm in diameter, ca. 1/2 the frond length, glabrous except at very bases; laminae ovate to deltate, 2–3 times pinnate (depending on size), mostly (12–)20–35 × (6–)15–20 cm, broadest at or just above the bases, tapering gradually to apices, costae (pinna axes) ascending mostly 30–50 degrees from rachis, pinnae acroscopically branched, basal acroscopic pinnule longer, more dissected, and at a greater angle with respect to costa than basal basiscopic branch (pinnae thus somewhat unequal-sided), laminae bluish green, often slightly glaucous; rachises castaneous to atropurpureous, glabrous, lustrous or slightly glaucous, terete to somewhat angled or obscurely sulcate adaxially (more so distally); pinnae of well developed laminae ca. 6–8 pinnate to bipinnate pairs below the 1-pinnate apical region (which is 3–5 cm long), decidedly alternate, stalked to ca. 2 cm (proximal pinnae); pinnulets (ultimate segments) obovate, flabellate, or rhombic, sometimes semicircular, non-articulate except on very old laminae (stalks not breaking cleanly, not cupule-like at their apex), mostly 1–2 × 1–2 cm (to 2 × 3 cm in sterile segments of juvenile fronds), cuneate at bases ca. 90–135 degrees (more narrowly cuneate bases on distal segments), occasionally pinnulet bases truncate and the pinnulets thus semicircular (especially proximal acroscopic pinnulet on well-developed pinnae), margins often cleft 1–4 mm, sterile pinnulets generally more incised than the fertile, 1–3 clefts per pinnulet, sterile margins thickened, whitish, shallowly crenulate distally, color of stalks gradually diminishing at base of pinnulets and passing slightly into pinnulet lamina; veins free, forking, in sterile laminae ending in small rounded crenulations (not in sharp teeth) of pinnulet margins, usually visible but only slightly raised, if at all, on both sides of laminae; indument lacking on both sides of laminae; idioblasts not apparent either abaxially or adaxially; sori 2–7 per pinnulet, confined to distal margins, sporangia borne on ± parallel veins on the strongly reflexed underside of the indusia (facing laminar tissue), mixed with sessile yellow-orange, cylindrical or clavate glands < 0.1 mm long; indusia mostly (1–)2–4 mm long, ca. 1 mm wide, entire or nearly so, oblong to slightly arcuate (not noticeably reniform), lacking hairs. 2n = 30 II.

#### Distribution and ecology.

**Figure 1.**
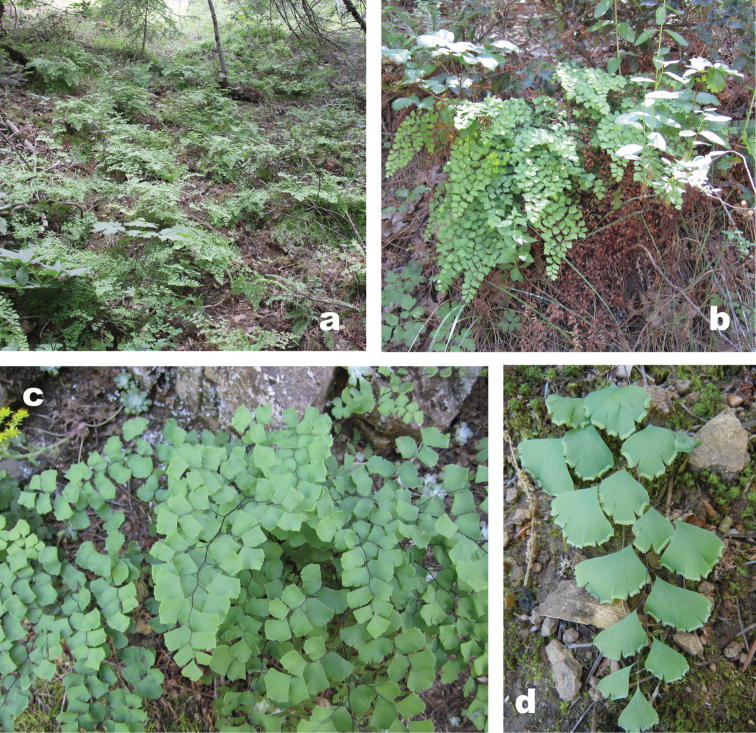
Photographs of *Adiantum shastense*. **A** Plants as dominant understory in one locality **B** Mature plant **C** Fronds **D** Young fertile pinnulets.

*Adiantum shastense* is currently known only from an area of the Eastern Klamath range (Miles and Goudy 1997) surrounding Shasta Lake entirely within Shasta County, California. It is found in mesic hardwood-conifer forests, on the forest floor as well as on limestone and metasedimentary rock outcrops, including rocky road cuts, most often in shade and with northern or eastern exposures. It co-occurs with the local endemic Shasta snow wreath, *Neviusia cliftonii* Shevock, Ertter & D.W.Taylor. In some localities it can be the dominant understory plant (Fig. 1a). Associated species include *Pinus ponderosa* P.Lawson & C.Lawson, *Quercus chrysolepis* Liebm., *Acer macrophyllum* Pursh, and *Toxicodendron diversilobum* (Torr. & A.Gray) Greene. Collected from 1100–2740 ft (335–835 m) elevation.

**Figure 2.**
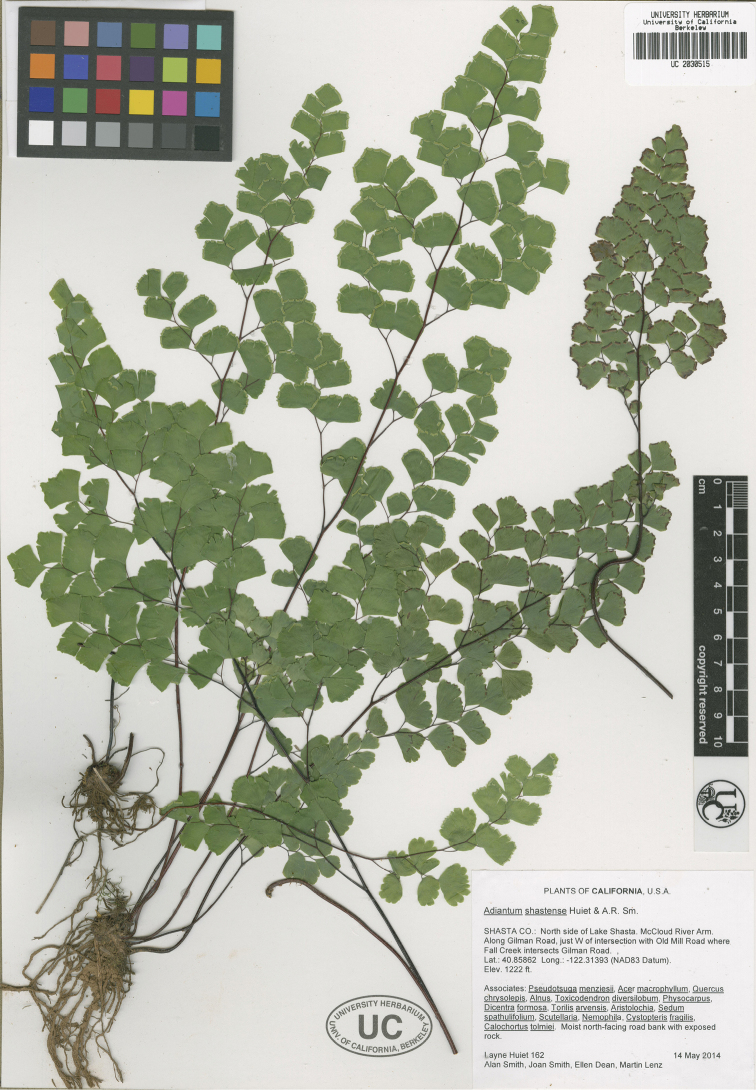
Holotype of *Adiantum shastense* Huiet & A.R.Sm. (*Huiet et al. 162*, UC)

#### Phenology.

Sporulates mid to late spring and most likely throughout summer.

#### Etymology.

It seems most appropriate to name this species *Adiantum shastense*, recognizing its restricted, currently known geographic localization to Shasta Co., California.

#### Suggested common name.

Shasta maidenhair fern.

#### Conservation status.

We recommend a California Rare Plant Rank of 4.3, a watch list for plants of limited distribution.

#### Paratypes.

U.S.A. California: Shasta Co.: 2.6 miles E of Nosoni Mountain on the west side of North Fork Squaw Creek, 2700 ft, 07 Apr 2009, *P.J. Alexander 994* (DUKE!, NMC, SP); Waters Gulch Trail ca. 05 mi N of Packers Bay Marina, 1200 ft, 27 Apr 1994, *Oswald & Ahart 6148* (CHSC, JEPS!); Bailey Cove Trail, W side of McCloud River arm of Shasta Lake, 1100 ft, 27 Apr 1994, *Oswald & Ahart 6121* (CHSC, JEPS!); Oak Run, 21 May 1894, *Baker & Nutting s.n*. (UC!); on limestone cliffs 0.5 mi E of Squaw Creek fire control station, 1750 ft, 19 Apr 1992, *Taylor 12599* (JEPS!); south side of Lake Shasta, Pit River Drainage along Fenders Ferry Rd, 1730 ft, 15 May 2014, *Huiet et al. 167* (DAV!, DUKE!, UC!); on arm of McCloud Reservoir across from boat ramp, ca. 8.5 air miles SSE of McCloud, 2740 ft, 27 May 2014, *Lenz & Nelson 5350* (DUKE!, UC!); along road to Deep Creek campground, ca. 5.2 air miles SE of Big Bend, 2395 ft, 27 May 2014, *Lenz & Nelson 5351* (DUKE!, UC!); along logging road on east side of Sacramento River across from Gibson, 2045 ft, 27 May 2014, *Lenz 5352* (DUKE!, UC!); McCandless Gulch ca. 5 miles E of Ingot, 1700 ft, 16 Sept 2014, *Taylor, Falscheer & Lindstrand 21512* (DAV!, UC!); Cedar Creek drainage, ca. 6 miles west of Round Mountain on Highway 299, 1450 ft, 6 Sept 2013, *Taylor 21418* (UC!); along Fender’s Ferry Rd. ca. 6.5 road miles SE of McCloud Bridge, 1800 ft, 3 Jul 2014, *Alverson 2014-10* (OSC, UBC, WTC!); north side of Lake Shasta, McCloud River Arm, Bailey Cove Trail, 1135 ft, *Huiet et al. 156A* (DUKE! chromosome voucher).

## Discussion

It was a surprise to discover that amongst the few herbarium collections of *Adiantum* from Shasta County, there was a previously unrecognized species. Before 2014, there were only 13 documented collections of *Adiantum* in the county (data from participants of the Consortium of California Herbaria, http://ucjeps.berkeley.edu/consortium). Amongst them were all the recognized taxa in California: *Adiantum aleuticum*, *Adiantum capillus-veneris*, and *Adiantum jordanii*. It was while confirming the identity of a recent collection that we serendipitously stumbled upon the new species. DNA sequencing analysis revealed that this plant was neither *Adiantum capillus-veneris* nor *Adiantum jordanii*, but rather sister to *Adiantum jordanii*. We confirmed that additional specimens collected near Shasta Lake gave the identical result (Huiet et al. unpublished). Subsequently, in spring 2014, we made collections from additional populations across a wider geographic range to confirm the earlier results and to examine material in the field.

Morphologically *Adiantum shastense* has rhizome scales that are essentially the same as in typical *Adiantum jordanii*. However the pinnulets of *Adiantum jordanii* are more fan-shaped and usually the sorus length is much longer so there are fewer sori per pinnulet than in *Adiantum shastense*. No doubt because of the shorter sorus length and the more cuneate shape to the pinnulets, *Adiantum shastense* has been identified by some as *Adiantum capillus-veneris*, the only other species found in California with similar blade architecture. However, the sori of *Adiantum shastense* have yellow farina among the sporangia, as does *Adiantum jordanii*. Also, the rhizome scales of *Adiantum capillus-veneris* are golden or lighter brown than those of *Adiantum shastense*. The most striking difference between *Adiantum shastense* and *Adiantum jordanii* is that *Adiantum shastense* is not ephemeral. Green laminae are persistent throughout the summer, and the fronds appear to overwinter without dying back, perhaps until more than a year’s persistence. After the fronds die back, they ring the base of the plant, surrounding the new growth.

The first collection of *Adiantum shastense* in Shasta County was over a century ago by Milo Baker and Frank Nutting, in 1894, and was identified by Baker as *Adiantum jordanii*. No other collection was made of *Adiantum shastense* until 1992 (*Taylor 12599*, UC) and that was identified as *Adiantum capillus-veneris*. Subsequent collections were mostly identified as *Adiantum capillus-veneris*. Thus far, the distribution of *Adiantum shastense* appears to be limited to a region surrounding Shasta Lake and the rivers and watersheds that feed into it and their drainages. A survey of other specimens identified as *Adiantum jordanii* and *Adiantum capillus-veneris* from nearby areas (counties) did not reveal any additional collections of *Adiantum shastense*. This includes a single collection of *Adiantum capillus-veneris* from Siskiyou county (UC), collected in a cave at Lava Beds National Monument (Smith et al. 1993). We also have examined specimens of *Adiantum jordanii* from Oregon and they too are correctly identified.

It appears that the Shasta maidenhair fern is another narrow endemic found in the area surrounding Shasta Lake. This region is host to a number of endemic plants and animals, most likely because of its unique geology, age and climate. These include the Shasta salamander, *Hydromantes shastae* Gorman & Camp, 1953 (Hammerson et al. 2004); the Shasta monkey flower, *Erythranthe taylori* Nesom (Nesom 2013); Shasta snow wreath, *Neviusia cliftonii* (Lindstrand and Nelson 2006); and the Shasta eupatory, *Ageratina shastensis* (D.W.Taylor & Stebbins) R.M.King & H.Rob. (Taylor and Stebbins 1993). Of these species, the Shasta maidenhair fern has one of the widest geographic ranges, perhaps due to its wind-borne spores. Currently we do not know if its range extends beyond the geologically unique Shasta Lake region into neighboring counties. Further field study may reveal more about this surprisingly new and unique California maidenhair fern. It currently is the only endemic species of *Adiantum* in the United States.

### Key to species of *Adiantum* in California

**Table:** 

1	Rhizomes stout, compact, short-creeping to suberect, usually 5–8 mm diam. (scales excluded); stipes mostly 1.5–3 mm diam. at bases; laminae palmate-pinnate (fan-shaped), proximal pinna pair 2–3-times basiscopically forked; pinnulets strongly inequilateral, 2–4 times longer than wide	***Adiantum aleuticum***
–	Rhizomes relatively narrow, short- to long-creeping (occasionally more compact in *Adiantum shastense*), usually 1.5–3 mm diam (scales excluded); stipes mostly 0.5–1.5 mm diam. at bases; laminae 2–3-pinnate (not fan-shaped), proximal pinna pair 0–1 times basiscopically forked; pinnulets more or less equilateral (bilaterally symmetric), about as long as wide or sometimes to twice as long as wide in *Adiantum capillus-veneris*.	
2	Rhizome and stipe base scales golden brown or light brown; laminae usually 2-pinnate; pinnulets (especially sterile ultimate segments) cut or lobed often >1/4 or much more than (to 2/3) the way to base; dark color of stalks extending into base of ultimate segments; distal teeth of sterile segments usually >3 mm long, acute at tips; pinnulet margins at base diverging at 45–90°; sori (and false indusia) (2–)3–11 per pinnulet, generally < 5 mm long	***Adiantum capillus-veneris***
–	Rhizome and stipe base scales dark brown or dark purplish brown; laminae 2–3-pinnate, larger fronds with proximal pinnae usually having at least 1 or 2 pairs of pinnules divided (i.e., laminae 3-pinnate proximally); pinnulets (especially sterile ultimate segments) cut or lobed usually <1/4 of the way to base; dark color of stalks extending into base of ultimate segments or ending ± abruptly at base of ultimate segments; distal teeth of sterile segments 1–2(–3) mm long, rounded or acute at tips; pinnulet margins at base diverging at 90–180(–240)°; sori (and false indusia) 1–5 per pinnulet, some generally > 5 mm long.	
3	Rhizomes short-to long-creeping, just below soil surface, stipe bases often > 5 mm apart; lamina tissue green; dark color of stalks ending ± abruptly at base of ultimate segments; ultimate segments often somewhat semi-lunate; mature fronds dying in late spring or early summer, completely dried and largely unseen in late summer, fall, and early winter; throughout California, but apparently rare in Shasta Co	***Adiantum jordanii***
–	Rhizomes short-creeping to suberect, often deeply buried, stipe bases < 5 mm apart; laminar tissue bluish green; dark color of stalks extending into base of ultimate segments; ultimate segments often somewhat rhomboidal; mature fronds persistent and evergreen through summer and into winter and following spring; Shasta Co., locally abundant	***Adiantum shastense***

Note: a combination of characters must occasionally be used to separate *Adiantum capillus-veneris*, *Adiantum jordanii*, and *Adiantum shastense*, i.e., not all characters are reliable for all specimens seen. However, the species characters for separating these three species are reliable for 95% or more of specimens seen. For example, *Gross 2802* (UC), from Ventura Co., is undeniably *Adiantum capillus-veneris* in rhizome characters, but blade characters resemble much more closely *Adiantum jordanii*. Pinnulet characters (shape, distal margin) often vary, depending on size of fronds and extent of fertility. Rare, sterile hybrids showing intermediate morphology are known between *Adiantum aleuticum* and *Adiantum jordanii* (Adiantum × tracyi C.C.Hall ex W.H.Wagner), but no known hybrids are known between other species. In California, and probably elsewhere, the four species have rarely been found growing together. In Shasta Co., *Adiantum aleuticum* and *Adiantum shastense* have so far only once been found growing proximate to one another, but *Adiantum jordanii*, does not co-occur with any of the other species, as far as we have observed.

## Supplementary Material

XML Treatment for Adiantum shastense
